# γ1-Containing GABA-A Receptors Cluster at Synapses Where they Mediate Slower Synaptic Currents than γ2-Containing GABA-A Receptors

**DOI:** 10.3389/fnmol.2017.00178

**Published:** 2017-06-08

**Authors:** Christine L. Dixon, Pankaj Sah, Angelo Keramidas, Joseph W. Lynch, Nela Durisic

**Affiliations:** ^1^Queensland Brain Institute, The University of QueenslandBrisbane, QLD, Australia; ^2^School of Biomedical Sciences, The University of QueenslandBrisbane, QLD, Australia

**Keywords:** γ1-subunit clustering, inhibitory neurotransmission, synapse, spillover, IPSC, artificial synapse, spt-PALM, uPAINT

## Abstract

GABA-A receptors (GABA_A_Rs) are pentameric ligand-gated ion channels that are assembled mainly from α (α1–6), β (β1–3) and γ (γ1–3) subunits. Although GABA_A_Rs containing γ2L subunits mediate most of the inhibitory neurotransmission in the brain, significant expression of γ1 subunits is seen in the amygdala, pallidum and substantia nigra. However, the location and function of γ1-containing GABA_A_Rs in these regions remains unclear. In “artificial” synapses, where the subunit composition of postsynaptic receptors is specifically controlled, γ1 incorporation slows the synaptic current decay rate without affecting channel deactivation, suggesting that γ1-containing receptors are not clustered and therefore activated by diffuse neurotransmitter. However, we show that γ1-containing receptors are localized at neuronal synapses and form clusters in both synaptic and extrasynaptic regions. In addition, they exhibit rapid membrane diffusion and a higher frequency of exchange between synaptic and perisynaptic populations compared to γ2L-containing GABA_A_Rs. A point mutation in the large intracellular domain and a pharmacological analysis reveal that when a single non-conserved γ2L residue is mutated to its γ1 counterpart (T349L), the synaptic current decay is slowed from γ2L- to γ1-like without changing the clustering or diffusion properties of the receptors. In addition, previous fast perfusion and single channel kinetic experiments revealed no difference in the intrinsic closing rates of γ2L- and γ1-containing receptors when expressed in HEK293 cells. These observations together with Monte Carlo simulations of synaptic function confirm that decreased clustering does not control γ1-containing GABA_A_R kinetics. Rather, they suggest that γ1- and γ2L-containing receptors exhibit differential synaptic current decay rates due to differential gating dynamics when localized at the synapse.

## Introduction

GABA type-A receptor (GABA_A_R) chloride channels mediate most of the fast inhibitory synaptic transmission in the mammalian brain. They are members of the pentameric ligand-gated ion channel family, and are assembled from 19 known subunits: α1–6; β1–3; γ1–3, δ, ε, π, θ and ρ1–3. The subunit composition determines the pharmacology, gating kinetics and subcellular localization of these receptors. More than half of all synaptic GABA_A_Rs contain the γ2 subunit, most often in the α1β2γ2 stoichiometry (McKernan and Whiting, 1996), as a result of which γ2-containing GABA_A_Rs have been studied intensely. However, much less is known about the functional properties of GABA_A_Rs containing γ1.

Although the γ1 subunit is weakly expressed throughout most of the brain, it is enriched in the central and medial amygdala, suggesting a physiological role in fear processing (Pirker et al., 2000; Esmaeili et al., 2009). Indeed, GABAergic inhibitory postsynaptic currents (IPSCs) with distinct pharmacological properties have been observed in central amygdala neurons (Delaney and Sah, 2001; Esmaeili et al., 2009). Given these observations, defining the physiological and pharmacological properties of γ1-containing synaptic GABA_A_Rs, and the mechanisms by which they are modulated, may provide insights into the function of the circuits in which these receptors are involved.

Using “artificial synapses” it has been shown that IPSCs mediated by α2β2γ1 GABA_A_Rs exhibit significantly slower rise and decay times than those mediated by α2β2γ2L GABA_A_Rs (Dixon et al., 2014). However, the inherent activation and deactivation rates for α2β2γ1 and α2β2γ2L GABA_A_Rs, when measured in macropatches using rapid agonist application or by single channel kinetic analysis, were similar (Dixon et al., 2014). Analysis of α2β2γ GABA_A_Rs incorporating chimeras of γ1 and γ2L subunits revealed that the intracellular loop and transmembrane domain 4 (TM4) regions of the γ1 subunit were responsible for the slow decay kinetics of α1β2γ1-mediated IPSCs. Because the same region of the γ2 subunit had previously been shown to be responsible for the synaptic localization of γ2-containing receptors (Nymann-Andersen et al., 2002; Alldred et al., 2005), we originally postulated that the slow decay rate of IPSCs mediated by α2β2γ1 GABA_A_Rs was due to reduced receptor clustering at synapses. Here, we use a combination of electrophysiology, super-resolution imaging and Monte Carlo simulations to investigate the mechanisms governing the IPSC decay kinetics, the synaptic localization and the mobility of γ1- and γ2L-containing GABA_A_Rs. We find that γ1-containing receptors exhibit slower synaptic current decay due to their differential inherent gating properties when localized at the neuronal synapse, rather than due to factors related to receptor mobility or proximity to the synapse.

## Materials and Methods

### Cell Culture

Our methods for generating artificial synapses have recently been published (Dixon et al., 2015). Briefly, E18 timed-pregnant rats were euthanized via CO_2_ inhalation in accordance with procedures approved by the University of Queensland Animal Ethics Committee. Cortical neurons were rapidly removed, plated onto poly-D-lysine-coated coverslips in a 4-well plate at a density of 80,000 cells/well, and cultured for 3–4 weeks until spontaneous IPSCs could be detected. Calcium phosphate was used to transfect HEK293 cells with GABA_A_R subunits (see below) together with empty pEGFP plasmid for cell identification, and neuroligin 2A to facilitate formation of synapses by the neurons onto the HEK293 cells. The transfection ratio was 1:1:4 for α2:β2:γ GABA_A_R subunits. One day post-transfection, the HEK293 cells were washed, trypsinized and plated on top of the neuronal cultures. Electrophysiological recordings were made 1–3 days after plating the cells. For imaging experiments, neuronal cultures were plated in a PDL-coated glass-bottomed dish at a density of 80–100 thousand cells per 29 mm dish. The transfection was performed 6–12 days after plating, and neurons were typically imaged between DIV 25 and DIV 28.

### Electrophysiology

Whole-cell recordings were obtained using a CsCl internal pipette solution (145 mM CsCl, 2 mM CaCl_2_, 2 mM MgCl_2_, 10 mM glucose, 10 mM HEPES, pH 7.4 CsOH). HEK293 cells were voltage clamped at −70 mV and perfused with a standard Ringer’s external solution (140 mM NaCl, 5 mM KCl, 2 mM CaCl_2_, 1 mM MgCl_2_, 10 mM HEPES, pH 7.4 NaOH) at the rate of 1 ml/min. Signals were digitized at 10 kHz and Bessel filtered at 4 kHz. Recording equipment was either a Axon Multiclamp 700B with Digidata 1440 and pClamp 10 recording software (Molecular Devices), or Axon Multiclamp 700A (Molecular Devices) with ITC-16 computer interface (Instrutech) and Axograph X recording software.

Except where otherwise stated, experiments were performed at room temperature (RT; 22.0 ± 1.5°C). Where increased temperature was required, an in-line bath heater (Warner TC-324B) was set using a bath thermistor to 32.0 ± 1.5°C.

Recordings with series resistance above 20 MΩ were discarded. Capacitance of the HEK293 cells was typically 20 pF, resulting in a typical corner frequency of 398 Hz. Because this was satisfactory for our experiments, series resistance compensation was not applied. Where drugs were applied, the events from the wash phase were averaged with the pre-drug period to control for any run-down or run-up.

Sliding templates in Axograph X were used to identify miniature events. Ten to ninety percent rise times were recorded and mono-exponential simplex fits applied to the decay period of individual events, which were then averaged for each cell.

### Drug Application

Frozen DMSO stocks of NO711 (Tocris) were diluted 1000-fold in extracellular solution on the day of recording. Drug was gravity fed through a 0.5 mm ID Teflon tube targeted at the cell being recorded, which was switched between control and drug solution using a two-way valve upstream of the bath (exchange time ~10 s). Background bath perfusion of control solution helped to minimize the drug effect on surrounding cells.

### Molecular Biology

In all experiments, we employed plasmid DNAs encoding human α2, β2, γ1 and γ2L GABA_A_R subunits, all in the pcDNA3.1 plasmid vector. The α2, β2 and γ1 or γ2L plasmids were co-transfected with pEGFP and neuroligin 2A (in pNICE) at a ratio of 1:1:4:1:1. In the high-resolution imaging experiments, the α2 and γ2L subunits were tagged near N terminus with superecliptic pHluorin (SEpH; Jacob et al., 2005; Tretter et al., 2008), a gift from Tija Jacob and Stephen Moss (Addgene plasmids 49169 and 49170). We used the existing AgeI and NheI enzyme sites to replace SEpH with the super-resolution protein mEos2 on the γ2L subunit, which was a gift from Frederic Meunier (Queensland Brain Institute).

Because both mEos2- and pHluorin-tagged γ2L constructs expressed well on the cell surface, we used them as a starting point for the construction of tagged γ1 subunits. We amplified the human γ1 subunit using Q5 polymerase (New England Biolabs) starting from amino acid 5 (forward primer XhoI γ1: CAAACTCGAGCTGGTTCCGCGTGGATCCGATGAAGATGATGAGGATTTAACGG, reverse primer γ1 SmaI GTAGCGTCACGTAGTCCCGGGGAATTTAGTCAGACTTCTTTTGATTTTTGC) and substituted it for the equivalent point on the γ2L subunit using existing SmaI and XhoI enzyme sites. The resulting constructs retained the linker and signal sequences of the original γ2L pHluorin subunit, thereby minimizing problems with surface expression, but the majority of the protein was γ1.

### Monte Carlo Simulations

Table 1 provides a list of default parameters used in the Monte Carlo simulations. Synapse geometry was defined using Cellblender 1.0 (MMBioS). In line with similar models (Montes et al., 2015), we used a simplified morphology with dimensions drawn from electron microscopy studies of synapses. The presynaptic bouton was represented by a box of dimensions 1 μm width, 1 μm depth and 0.5 μm height. Below this, the postsynaptic cell membrane was modeled as a 2-dimensional plane, 64 μm by 64 μm. The plane was positioned 20 nm below the surface of the presynaptic bouton, consistent with estimates of the synaptic cleft size. A total of 300 postsynaptic receptors were inserted into the plane and distributed across an area of 200 nm by 200 nm. These dimensions are based on the larger end of the range of GABAergic synapses in brain tissue (Nusser et al., 1997; Kasugai et al., 2010). At the initiation of the simulation, 3000 molecules of neurotransmitter were released from a point source 1 nm below the center of the presynaptic terminal. In subsequent simulations, we increased the postsynaptic area without changing the number of receptors, to represent reduced receptor clustering that may be occurring in γ1-containing artificial synapses (Table 2). As detailed below, we also systematically varied other parameters. A data-derived Markov model from our previous work was initially used to define receptor-agonist interactions and receptor opening; α1β2γ2 receptor kinetics were used to test the hypothesis because more rapid waveforms reduced simulation times and because our previous experiments showed that the slow kinetics associated with γ1 subunits are independent of which α subunit was expressed (Dixon et al., 2014). To ensure that our results were not model-dependent, we repeated the simulation of increased clustering with a very different Markov scheme, incorporating mono-liganded channel openings co-operative binding and desensitization (Petrini et al., 2011). The fastest realistic diffusion rate for an amino acid in an aqueous environment is 0.76 μm^2^/ms (Longsworth, 1953). However, narrow tortuous spaces and extracellular matrix molecules impede diffusion considerably. As neurotransmitter mobility when diffusing through neuropil is thought to be at least 2.5-fold slower than in free solution (Rusakov et al., 2011), we chose a baseline diffusion rate of 0.3 μm^2^/ms. Simulations were run in mCell3.3 (MMBioS), using 20–50 consecutive seed values, which were averaged. Mono- or bi-exponential decay fits were carried out using Graphpad Prism on the averaged events.

**Table 1:** Monte Carlo simulation parameters.

Parameter	Starting value	References
Number of channels	300	Nusser et al. (1997)
Synaptic cleft width	20 nm	Schikorski and Stevens (1997), Zuber et al. (2005)
GABA molecules per vesicle	3000	Clements (1996)
GABA diffusion rate	0.3 μm^2^/ms	Nielsen et al. (2004)
Receptor gating kinetics	α1β2γ2	Dixon et al. (2014)
Width of PSD	200 nm	Kasugai et al. (2010)

*The intrinsic receptor parameters were taken from a kinetic model for α1β2γ2L GABA_A_Rs presented in Dixon et al. (2014)*.

**Table 2:** Summary of Monte Carlo simulation results.

Experiment	Diffusion (μm^2^/ms)	Cleft (nm)	Cluster (nm)	Peak (# open receptors)	Decay tau (ms)
Changing synaptic area	0.3	20	200	26.5	17.1
	–	–	400	16.1	16.8
	–	–	800	6.8	17.0
	–	–	1600	1.9	15.8
Changing diffusion constant	0.1	20	200	68.5	18.5
	0.3	–	–	26.5	17.1
	0.8	–	–	8.0	17.2
Changing synaptic cleft	0.3	10	200	52.0	17.0
	–	15	–	35.8	17.5
	–	20	–	26.5	17.1
5 release events 1 ms apart	0.3	20	200	94	25
	–	–	400	76.7	27
	–	–	800	41.9	28
	–	–	1600	12.1	28
Changing synaptic area (α1β1γ2)	0.3	20	200	194.3	79.6
	–	–	400	156.7	79.5
	–	–	800	92.9	81.6
	–	–s	1600	27.9	82.3

*In addition to receptor spread and cleft size, GABA diffusion coefficient was systematically increased to reflect the change of neurotransmitter diffusion with temperature. All results were averaged from 20 to 50 simulations of GABA release onto α1β2γ2L GABA_A_Rs, except where α1β1γ2 receptors are specified. Reported time constants are from mono-exponential fits, except for α1β1γ2 receptors where weighted time constants from bi-exponential fits are reported*.

### Microscopy Experiments

All experiments were performed on an Elyra PS1 STORM/SIM microscope (Carl Zeiss Microscopy GmbH) equipped with a 100× objective (α Plan-Apochromat 100× 1.46 NA oil-immersion), a focus lock system and an EMCCD camera Andor iXon Ultra 897 (Andor Technologies). A LF488/561-A-000 beam splitter and a FF01–523/610–25 emission filter (Semrock) were used to record SEpH and mEos2 fluorescence. Antibodies against GFP labeled with Alexa 647 and anti-synaptotagmin antibodies labeled with Oyster 550 or Oyster 650 (Synaptic Systems) were detected using LF 405/488/561/635-A-000-ZHE and FF01-446/510/581/703-25 (Semrock). In dual color experiments, red and green channels were aligned with fluorescent microspheres (Molecular Probes) and the channel alignment module in Zen 2012 SP2 (black) software (Carl Zeiss Microscopy GmbH). We collected movies of labeled synaptotagmin sites in live neurons and only bright immobile fluorescent puncta were used to determine presynaptic terminals. Synaptic GABA_A_Rs were then identified from the overlap of synaptotagmin-rich presynaptic terminals with GFP-labeled γ subunit clusters. Cells were imaged in total internal reflection (TIRF) or highly inclined illumination mode in an enclosed chamber at RT, (32.0 ± 1.5)°C or (36.0 ± 1.5)°C.

### sptPALM

For single particle tracking super-resolution photoactivation localization microscopy (sptPALM) measurements were performed as previously described (Manley et al., 2010). A 560 nm laser was adjusted to 9–14 W/cm^2^. mEos2 molecules were continuously converted with a 405 nm laser, the power of which was gradually increased. This ensured that only single mEos2 molecules were converted and imaged at 560 nm while keeping their number constant, and well separated during the acquisition. The density of fluorescent particles was kept below 0.2 molecules/μm^2^. For each cell expressing α2β2γ1 GABA_A_Rs, image stacks containing 2000–4000 images were collected at 40 Hz. Cells expressing α2β2γ2 were imaged at either 20 or 40 Hz. Both sampling rates resulted in the same diffusion coefficient.

### uPAINT

Universal point accumulation imaging in nanoscale topography (uPAINT) experiments were performed as previously described (Giannone et al., 2010). We initially conducted two sets of experiments. In one, we used fluorescent nanobodies against GFP and in another, we used Alexa647-conjugated anti-GFP antibodies. Fluorescently tagged antibodies against GFP can accommodate more fluorophores and therefore yield longer trajectories, which significantly increases the accuracy of diffusion coefficients calculated from individual trajectories (Ernst and Köhler, 2013). However, they can cause cross-linking at higher concentrations. We compared diffusion parameters from these two experiments to determine an optimal concentration of anti-GFP antibodies at which single GABA_A_Rs were labeled and potential cross-linking of the receptors was negligible. Cells were imaged in oblique illumination mode with a 642 nm laser at 20–30 W/cm^2^.

### Data Analysis: sptPALM and uPAINT

Single molecule fluorescent spots were localized in each frame and their position was followed over time using the TrackMate tracking algorithm (Jaqaman et al., 2008). A custom written MatLab (Math Works) routine was used to visualize individual trajectories and their corresponding mean-squared displacement (MSD) plots and to generate an average MSD vs. lag time plot. To improve the relative statistical error of individual MSD curves, only trajectories with 20 points or more were included in the analysis. It has previously been shown that when the diffusion coefficient is calculated from a linear fit to the first four points of the MSD vs. time plot, the relative error of the fit increases as the number of points decreases. As a consequence, the size of the diffusion coefficient distribution increases and the range of measured diffusion coefficients can still differ by a factor of two. In such cases the best estimate for the diffusion coefficient corresponds to the mean value (Qian et al., 1991; Ernst and Köhler, 2013). We therefore calculated the diffusion coefficients from the mean MSD vs. time plot of all trajectories recorded per cell.

Diffusion coefficient (D) was calculated by fitting the first four points of the average MSD curve to the equation:

(1) MSD(t) = 4Dt + b

An average confinement size was calculated by fitting MSD vs. time plot to the equation:

(2) $$MSD\left(t\right)\;=\;\frac{L^{\text{2}}}{3}\left(1\;-\;\exp\left(-\;\frac{12Dt}{L^{\text{2}}}\right)\right)\;+\;4D_{mac}t,$$

where *L*^2^ is confinement area in which diffusion is constricted and *D*_mac_ is diffusion coefficient at long timescale. An average localization precision if fluorescence spots was 40–60 nm.

### Statistical Analyses

Statistical significance was evaluated with a Dunnet’s multiple comparisons test, two-way analysis of variance (ANOVA) or Student’s *t*-test as indicated in the text. The tests were run within GraphPad Prism or SigmaPlot computer programs. All data are presented as mean ± SEM from *n* cells and a minimum of two independent experiments.

## Results

### Electrophysiological Analysis of IPSCs in Artificial Synapses

**Figure 1:**
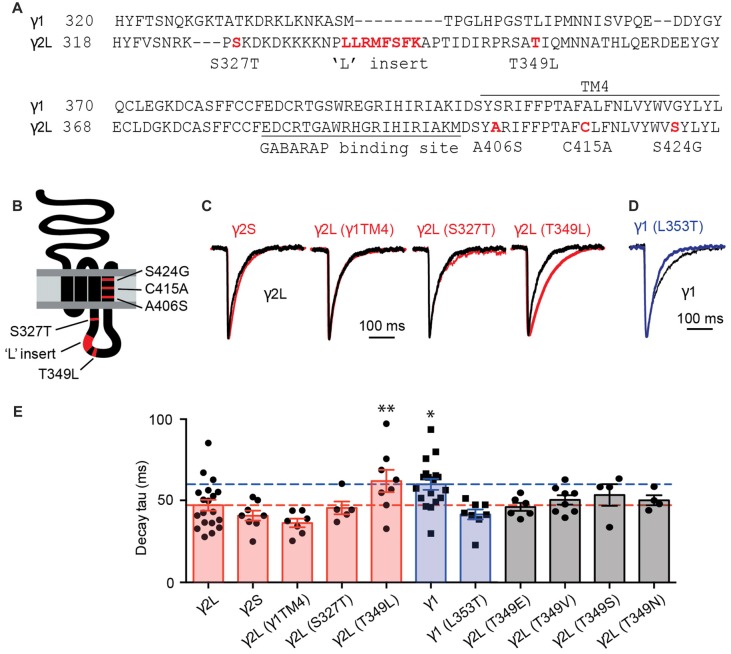
The inhibitory postsynaptic current (IPSC) decay time constant was increased by the T349L mutation to the γ2 subunit and decreased by the converse (L353T) mutation to the γ1 subunit. **(A)** Amino acid sequence alignment of the large intracellular and transmembrane domain 4 (TM4) domains of the γ1 and γ2 subunits. Non-conserved residues or regions that were investigated in this study are highlighted in red. **(B)** Membrane topology of a γ2 subunit indicating the location of residues and domains highlighted in red in **(A)**. **(C)** Average waveforms from individual representative HEK293 cells that expressed α1β2γ2L GABA-A receptors (GABA_A_Rs; black) or α1, β2 and the indicated γ2 subunit variant (red). **(D)** Average waveform from a representative HEK293 cell that expressed α1β2γ1 GABA_A_Rs (black) compared with a HEK293 cell expressing α1β2γ1^L353T^ GABA_A_Rs (blue). **(E)** Mean decay time constants recorded from artificial synapses incorporating the indicated γ subunit variant. Individual data points represent the average of all IPSCs recorded in a single cell and error bars indicate the SEM. Bar plots are color coded according to whether they represent γ2L to γ1 subunit changes (red), γ1 to γ2L subunit changes (blue) or γ2L T349 phosphorylation site mutations (gray). Some of the data from γ2L- and γ1-containing receptors have previously been published (Dixon et al., 2014). Horizontal lines indicate mean decay time constants for γ2L (red) and γ1 (blue). *P < 0.05, **P < 0.01 relative to γ2L by one-way analysis of variance (ANOVA) followed by Dunnett’s *post hoc* test for multiple comparisons.

Using artificial synapses, it has been shown that spontaneous IPSCs mediated by α2β2γ1 GABA_A_Rs exhibited slower decay rates than those mediated by α2β2γ2L GABA_A_Rs (Dixon et al., 2014). By analyzing chimeras constructed of γ2L and γ1 subunits, it has been concluded that the TM4 region and the intracellular loop of γ2L were required for the faster IPSC kinetics (Dixon et al., 2014). In the current work, we focused on this region in an attempt to identify which residues are responsible for functional differences between GABA_A_Rs containing γ1 and γ2L subunits. An amino acid sequence alignment of the relevant regions is displayed in Figure 1A and a schematic of the γ subunit is shown in Figure 1B, with key non-conserved regions of the TM4 and intracellular domains highlighted in red.

Three TM4 residues were found to differ between γ1 and γ2L subunits, so we investigated the possibility that these were responsible for the slow decay rate (and possible loss of synaptic clustering) of α2β2γ1 GABA_A_Rs. All three γ2L residues were mutated to their γ1 counterparts and the resulting triple mutant subunit, designated γ2L(γ1TM4), were co-expressed with α2 and β2 subunits in HEK293 cells that were plated onto cultured neurons to form synaptic contacts (see “Materials and Methods” Section). IPSCs mediated by α2β2γ2L(γ1TM4) GABA_A_Rs exhibited a mean decay time constant of 36.4 ± 2.6 ms (*n* = 7), similar to that of wild-type α2β2γ2L GABA_A_Rs (47.3 ± 3.3 ms, *n* = 19) but faster than that of wild-type α2β2γ1 receptors (60.1 ± 3.4 ms, *n* = 18; Figures 1C,E). Next, we tested several non-conserved regions in the intracellular loop that are known to have functional importance in γ2 subunits. The first of these was the γ2L splice variant, which incorporates eight amino acids that are absent from both the short splice variant (γ2S) and γ1 (Figure 1A). GABA_A_Rs incorporating the γ2S variant are differentially expressed across different brain regions, and have been reported to exhibit reduced synaptic clustering relative to receptors containing the γ2L isoform (Meier and Grantyn, 2004). We found that the decay time constant for γ2S-containing receptors (41 ± 3 ms; *n* = 8) was indistinguishable from that of γ2L-containing receptors (Figures 1C,E). This is consistent with the observation that transgenic mice suffer no deficit when only γ2S or γ2L is expressed (Baer et al., 2000). The rise times of synaptic currents are sensitive to the GABA concentration profile and can reflect changes in clustering propensity. We therefore calculated IPSC 10%–90% rise times for the γ subunit variants listed above and found that γ1-containing receptors have slower rise times than γ2-containing GABA_A_Rs (Supplementary Figure S1A).

GABA_A_Rs have a number of phosphorylation sites that modulate their trafficking and their ability to bind to other proteins (Nakamura et al., 2015). Protein kinase C phosphorylates γ2L at Ser 327 (Moss et al., 1992), and de-phosphorylation of this site leads to decreased GABA_A_R clustering at synapses (Muir et al., 2010). We therefore investigated expression of receptors in which the phosphorylated residue in γ2L had been mutated to the γ1 equivalent. Mutation of serine 327 (α2β2γ2L^S327T^) resulted in IPSCs with a mean decay time constant of 45.6 ± 3.9 ms (*n* = 5), similar to wild-type α2β2γ2L GABA_A_Rs (Figures 1C,E). Mutation of a second putative phosphorylation site, Thr 349 (α2β2γ2L^T349L^), resulted in IPSCs with a mean decay time constant of 62 ± 6.8 ms (*n* = 8), much closer to the decay time constant seen with the α2β2γ1 GABA_A_Rs, and significantly different from that of wild-type α2β2γ2L GABA_A_Rs (*p* = 0.04, Dunnett’s multiple comparisons test). Converse mutation at the corresponding residue in the γ1 subunit, α2β2γ1^L353T^, resulted in IPSCs with a faster decay time constant (41.7 ± 3.1 ms, *n* = 8; Figures 1D,E). A two-way ANOVA comparing the results of wild-type γ1 and γ2L containing GABA_A_Rs to those containing the γ1^L353T^ and γ2L^T349L^ subunits showed that the residue at this position determined the IPSC decay time constant (*p* = 0.0004). The same analysis performed for rise time did not show a significant effect (*p* = 0.33). This demonstrates that two amino acids, γ2L Thr349 and γ1 Leu353, are critical in setting the decay kinetics of IPSCs containing these subunits.

We next sought to determine whether phosphorylation of γ2L Thr349 also induces a slowing in IPSC decay rate. CaMKII is absent from HEK293 cells (Houston and Smart, 2006), although these cells do express other kinases that would act as a substitute (Zagranichnaya et al., 2005). We employed a mutagenesis approach to investigate this question, first substituting γ2L Thr349 with negatively charged glutamate as a phosphomimetic. We found that IPSCs mediated by α2β2γ2L^T349E^ GABA_A_Rs had similar kinetics (46.3 ± 2.5 ms, *n* = 6) to those mediated by wild-type α2β2γ2L GABA_A_Rs (Figure 1E). This could indicate either that phosphorylation is not involved in this phenomenon, or that γ2L Thr349 is a target for a kinase endogenous to HEK293 cells, and is phosphorylated at baseline. However, if this was true, then substituting with the non-polar, non-phosphorylable valine should make the events more similar to those observed in α2β2γ1 GABA_A_Rs, which was not the case; events from synapses containing α2β2γ2L^T349V^ GABA_A_Rs exhibited a decay time constant of 50.6 ± 2.9 ms (*n* = 8). We also investigated the conservative Ser and Asn mutations and found no significant change in decay time constants (Figure 1E). Taken together these results lead us to conclude that the difference in IPSC decay rate between α2β2γ1 and α2β2γ2L GABA_A_Rs is not driven by phosphorylation of the γ2 subunit.

**Figure 2:**
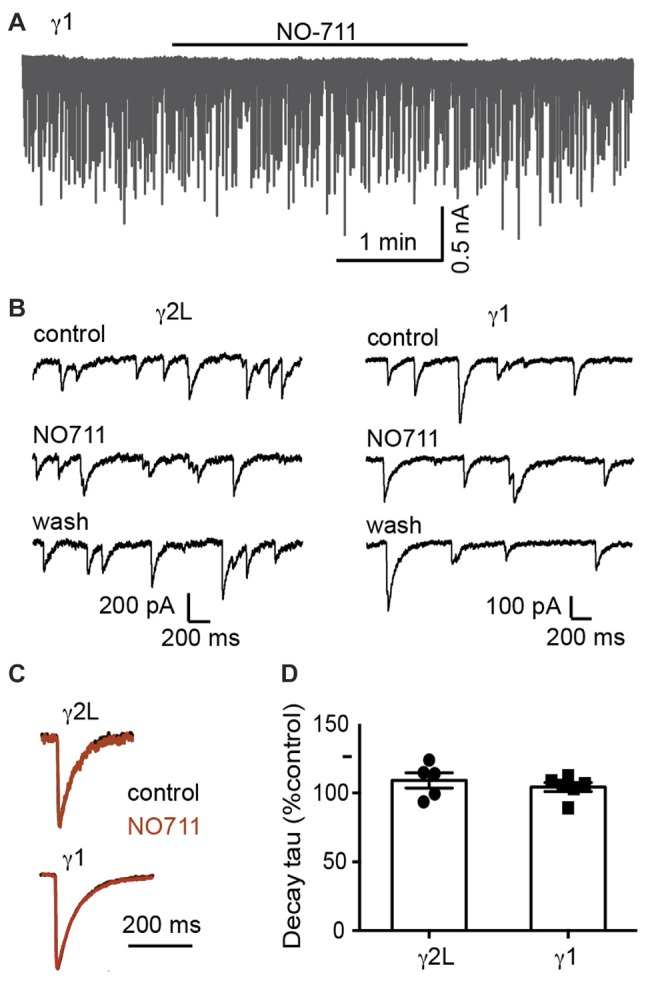
Blocking GABA reuptake did not change the IPSC decay time constant. **(A)** Sample recording of spontaneous IPSCs in a co-cultured HEK293 cell expressing α2β2γ1 GABA_A_Rs, showing no obvious effect of 10 μM NO-711. **(B)** Examples of recordings at higher temporal resolution of co-cultured HEK293 cells expressing α2β2γ2L and α2β2γ1 GABA_A_Rs, before, during and after 10 μM NO-711 application. **(C)** Averaged IPSC waveforms from individual cells, before and during drug application. **(D)** Quantification of percentage change in IPSC decay time constant during NO-711 application, normalized to pre-drug and wash values.

We next sought to understand the mechanism underlying the difference in IPSC decay rates between α2β2γ1 and α2β2γ2L GABA_A_Rs. One possibility is that α2β2γ1 GABA_A_Rs are less clustered than α2β2γ2L GABA_A_Rs (Dixon et al., 2014), in which case the slower kinetics of α2β2γ1-mediated IPSCs could be caused by the delay in neurotransmitter reaching the de-clustered, peri-synaptic receptors. Synaptically released GABA is cleared by a combination of diffusion and uptake by the GABA transporter, GAT1. *In situ*, it is clear that the transporter is critical, reducing the spread of GABA and activation of peri-synaptic receptors (Gonzalez-Burgos et al., 2009). We therefore investigated whether blocking GAT1 differentially affected IPSCs mediated by α2β2γ1 and α2β2γ2L GABA_A_Rs. However, blocking GAT1 activity with the specific blocker, NO711 (10 μM), had no impact on the kinetics of IPSCs mediated by either receptor (Figure 2). Decay time constants for α2β2γ2L-mediated IPSCs were 109 ± 6% of control (*n* = 5), and for α2β2γ1-mediated IPSCs were 104 ± 3% of control (*n* = 6).

**Figure 3:**
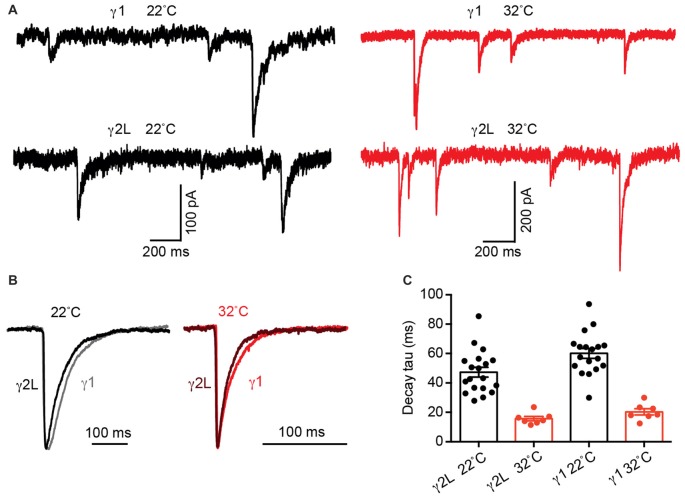
Increasing temperature accelerated α2β2γ2L- and α2β2γ1-mediated IPSC decay time constants to an equivalent extent. **(A)** Sample recordings of IPSCs at 22 and 32°C from co-cultured HEK293 cells expressing α2β2γ2L and α2β2γ1 GABA_A_Rs showing increased amplitude and accelerated IPSC decay at higher temperature. **(B)** Averaged IPSC waveforms from indicated receptors at 22°C and 32°C. **(C)** For both receptor types, the mean IPSC decay time constant was accelerated three fold by the 10°C temperature increase, indicating that the difference in the IPSC decay rate is dominated by the receptor gating properties and not by the GABA diffusion rate.

The intrinsic deactivation rates of α2β2γ1 are identical to those of α2β2γ2L GABA_A_Rs when expressed in a non-synaptic context (Dixon et al., 2014). If the difference in IPSC decay rate is determined by differences in molecular interactions (i.e., gating conformational changes) within the protein, the kinetics of channel deactivation should be temperature sensitive, with a Q10 > 2 (Postlethwaite et al., 2007). In contrast, if differences in the neurotransmitter diffusion rate control the IPSC decay rate difference, the temperature sensitivity should exhibit a reduced Q10 of 1–2 (Wahl et al., 1996). We therefore tested the temperature sensitivity of IPSCs mediated by α2β2γ1 and α2β2γ2L GABA_A_Rs at 22 and 32°C (Figure 3A). At 32°C, α2β2γ1-mediated IPSCs exhibited an average decay time constant of 20.3 ± 2.1 ms (*n* = 7), whereas for α2β2γ2L-mediated IPSCs this value was 15.7 ± 1.5 ms (*n* = 7). When these data were compared to the respective values obtained at 22°C, a Q10 value of 3 was obtained for both receptor isoforms (Figures 3B,C). A two-way ANOVA showed that temperature was a key determinant of decay the time constant (*p* < 0.0001), as was receptor type, in line with previous results (*p* = 0.03). IPSC rise times follow similar trend (Supplementary Figure S1B). From this, we infer that the difference in IPSC decay rate between the two subtypes is dominated by differential molecular interactions (i.e., gating conformational changes) when in the synapse and not by diffusion of GABA.

### Monte Carlo Simulations

**Figure 4:**
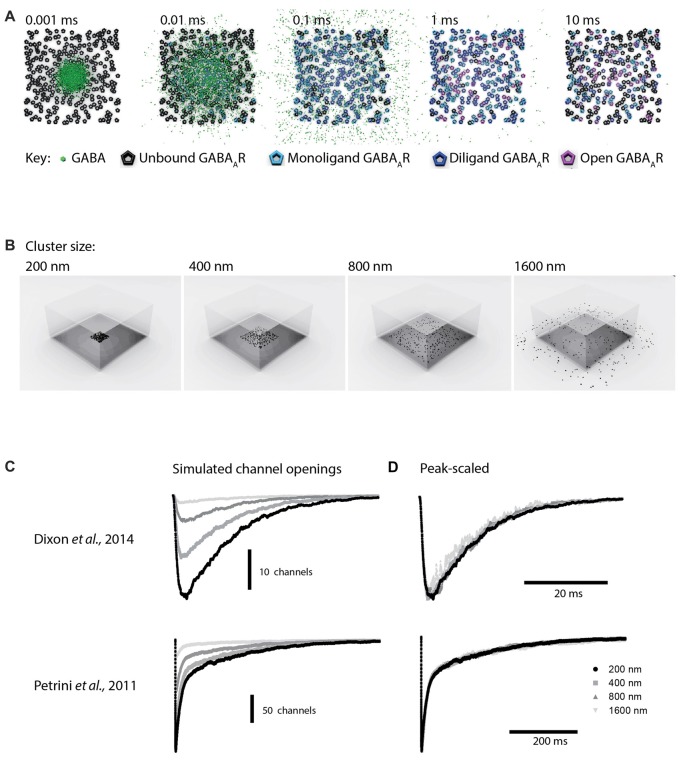
Monte Carlo simulations of synaptic function. The default parameters used to set up the simulations are provided in Table 1.** (A)** Top view of a simulation of the postsynaptic density with the presynaptic terminal removed. A total 300 receptors are spread across a 200 × 200 nm square, and 3000 GABA molecules were released from a point source at time 0. To illustrate the time required for GABA to leave the cleft, the slowest diffusion constant we tested (0.1 μm^2^/ms) is shown. Diffusion of GABA out of the synaptic cleft occurs rapidly, within 0.01 ms, meaning that very few if any extrasynaptic binding events are likely to slow the decay time of IPSCs. **(B)** The same model, with the 300 receptors spread out across increasingly large postsynaptic densities. The presynaptic terminal is transparent.** (C)** Sample waveforms generated by the simulation shown in **(B)** with a realistic diffusion constant of 0.3 μm^2^/ms. Peak scaled waveforms show that the IPSC decay time course is unaffected by the receptor clustering density. **(D)** As for **(C)**, but this time using an alternative receptor model that included mono-liganded openings and co-operative agonist binding.

To more closely examine effects of synaptic spillover on IPSC kinetics, we turned to Monte Carlo simulations of synapse function. A list of the initial default parameters used to generate a model of a functional synapse is given in Table 1 and the kinetic schema are shown in Supplementary Figure S2A. Initially, the synapse contained a fixed number of receptors (300) spread out over an increasing postsynaptic surface area. Snapshots taken at indicated time intervals during one such simulation are shown in Figure 4A. With a postsynaptic area of 200 nm by 200 nm, a single vesicle of transmitter activated 69 GABA_A_Rs, and the evoked IPSC exhibited a rise time of 1.6 ms and a decay time constant of 12.9 ms, similar to the results obtained in artificial synapses (Dixon et al., 2014). With the same receptors spread out over a larger area (400 × 400 nm), the number of activated GABA_A_Rs decreased to 51, and the evoked IPSC had a rise time of 1.7 ms and a decay time constant of 12.7 ms. This trend towards a reduced number of open receptors with little change in synaptic kinetics continued with the increase of postsynaptic surface areas (Figures 4B,C). Systematic alterations in the GABA diffusion rate, which would be caused by temperature changes *in vivo*, or in the width of the synaptic cleft did not change this relationship (Table 2). Similarly, extending the synaptic release time course, by simulating the release of five vesicles at 1 ms intervals, had no effect on this relationship (Table 2). Finally, we again tested the impact of receptor spread but this time used a very different receptor model, in which monoliganded openings caused a decrease in decay rate when receptors are located more diffusely (Supplementary Figure S2B; Petrini et al., 2011). However, this model also failed to show that reduced clustering could be a plausible mechanism for slow decay times at α2β2γ1-containing synapses (Figure 4D).

### Single-Particle Tracking of GABA_A_Rs in HEK293 Cells

Ligand-gated ion channel receptors can move rapidly between extrasynaptic regions and the synaptic stabilization zone (Gerrow and Triller, 2010; Choquet and Triller, 2013; Salvatico et al., 2015). Changes in diffusion dynamics often occur within receptor clusters, and depend on receptor subtype, phosphorylation status and interactions with other proteins (Muir et al., 2010; Muir and Kittler, 2014; Lévi et al., 2015). To test if there is a link between GABA_A_R membrane stability and IPSC kinetics, we employed single-particle tracking to follow individual receptors containing γ1^mEos2^, γ2L^mEos2^ or γ2L^T349L-mEos2^ subunits in real time. Fusion of the mEos2 fluorescent protein to the γ subunit extracellular domain allowed us to observe the ensemble of receptors in the green channel and to track the movement of individual receptors after converting mEos2 to its red form. In addition, in a subset of experiments we also tagged the α2 subunit with the pH-sensitive GFP (SEpH); this allowed us to visualize the receptors in green even after mEos2 had been converted to its red form.

**Figure 5:**
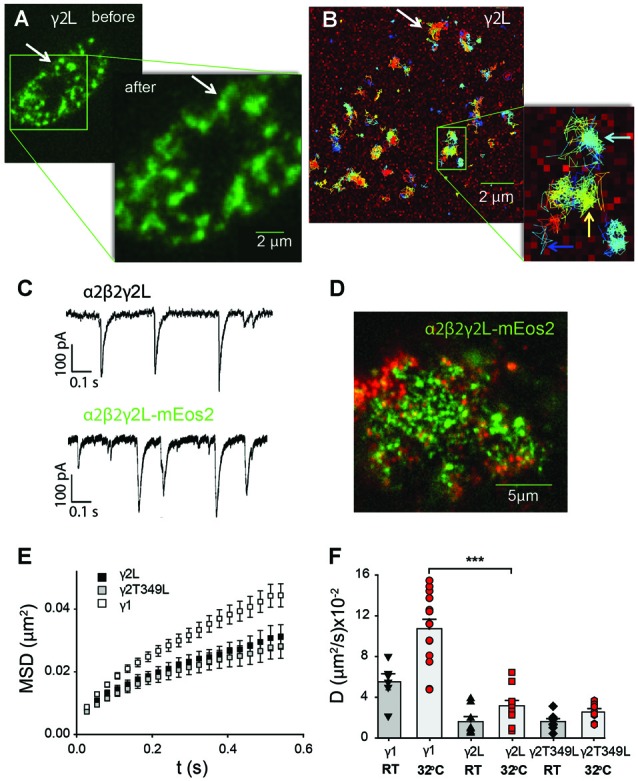
Single-particle tracking of GABA_A_Rs expressed non-synaptically in HEK293 cells. **(A)** Total internal reflection (TIRF) image of the bottom membrane of a HEK293 cell transfected with α2^SEPH^β2γ2L^mEos2^ GABA_A_Rs and imaged before and after the single-particle tracking experiment shown in **(B)**. The GABA_A_Rs are organized in domains whose restructuring occurs over several minutes (white arrow). **(B)** Single-particle tracking photoactivation localization microscopy (sptPALM)trajectories of single GABA_A_Rs show that their movement is confined within domains (light blue arrow). A small number of channels can move between domains (yellow arrow) or diffuse freely (dark blue arrow). For clarity only a subset of trajectories is shown. **(C)** An example of spontaneous synaptic currents recorded from a HEK cell in co-culture expressing α2β2γ2L-mEos2. The receptors formed with labeled γ2L subunit are functional and their synaptic currents are similar to those of wild-type receptors. **(D)** GABA_A_Rs (green) are only partially co-localized with endogenous gephyrin (red) in HEK 293 cells. **(E)** Averaged (±SEM) mean-squared displacement (MSD) vs. time plots for receptors incorporating the indicated mEos2-tagged γ subunits recorded at room temperature (RT). All MSD curves reflect confinement of receptors within nanodomains. **(F)** Average diffusion coefficient of receptors containing mEos2-tagged γ1, γ2 and γ2L^T349L^ subunits measured at RT or 32°C. ***P < 0.001.

The splitting, merging and reshaping of receptor clusters could be followed on the scale of minutes, and did not depend on the receptor isoform. Figure 5A shows a snapshot of receptor distribution on the cell membrane of a HEK293 cell transfected with α2^SEpH^β2γ2L^mEos2^ GABA_A_Rs. The inset shows another snapshot taken 2 min later, where two representative clusters (arrow, Figure 5A) can be seen to have merged. It is apparent that single receptors are mobile over that time period and move between both clusters (white arrow in Figure 5B). In addition, single GABA_A_Rs also moved within distinct regions as suggested by the many clusters that remained unaltered (colored arrows in in Figure 5B). Addition of fluorescent protein to the γ subunit did not change the decay rates of spontaneous IPSCs mediated by GABA_A_Rs (Figure 5C). In neurons, GABA_A_Rs containing the γ2L subunit interact with the scaffolding protein gephyrin, which is also endogenously expressed in HEK293 cells. However, in HEK293 cells we only observed limited co-localization of α2β2γ2L receptors and gephyrin (Figure 5D).

Overall, γ1-containing receptors were more mobile than those containing γ2L subunits. At 32°C the fraction of α2β2γ1^mEos2^ GABA_A_Rs undergoing free diffusion or changing domains was 28 ± 4%, compared to only 12.0 ± 0.4% of α2β2γ2L^mEos2^ GABA_A_Rs (over 2000 trajectories from a minimum of 10 cells per transfection type). GABA_A_Rs containing the T349L mutation in the γ2L subunits behaved similarly to α2β2γ2L^mEos2^ GABA_A_Rs and had 11.7 ± 0.4% of the trajectories located outside of single domains. Single GABA_A_R trajectories were further analyzed in terms of MSD to determine the characteristic diffusion coefficient. Figure 5E shows representative MSD vs. time plots for the different receptor subunit combinations. All MSD plots exhibited a negative deviation from a straight line, indicating confinement of the receptors in the subdomains.

At RT, the average diffusion coefficient for α2β2γ2L^mEos2^ GABA_A_Rs was (1.6 ± 0.5) × 10^−2^ μm^2^/s (Figure 5F), whereas the diffusion coefficient of α2β2γ1^mEos2^ GABA_A_Rs was (5.5 ± 0.8) × 10^−2^ μm^2^/s, which is significantly higher (Student *t*-test, *P* < 0.0001). Moreover, the α2β2γ2L^T349L/mEos2^ GABA_A_Rs, which exhibit similar IPSC decay rates to α2β2γ1 GABA_A_Rs, diffused at a similar rate to the α2β2γ2L^mEos2^ GABA_A_Rs, with a mean diffusion coefficient of (1.6 ± 0.3) × 10^−2^ μm^2^/s. With the increase in temperature to 32°C, the mobility of single receptors increased 1.9-fold for α2β2γ1 and α2β2γ2L GABA_A_Rs; 10.7 ± 0.9) × 10^−2^ μm^2^/s and (3.5 ± 0.1) × 10^−2^ μm^2^/s, respectively) and 1.5 times for α2β2γ2L^T349L^ GABA_A_Rs (to a value of (2.6 ± 0.3) × 10^−2^ μm^2^/s; Figure 5F). These values are similar to those previously reported for α2-containing GABA_A_Rs in hippocampal neurons (Hausrat et al., 2015).

Because our sptPALM experiments were performed in the TIRF configuration with the penetration depth ranging from 100 nm to 120 nm, it is possible that some of the imaged GABA_A_Rs may have been located intracellularly. To address this, we replaced mEos2, which was inserted near the N-terminus in the extracellular part of the γ subunit, with SEpH and used Alexa 647 conjugated anti-GFP antibodies for single-particle tracking in uPAINT experiments (Giannone et al., 2010). This method ensured that we imaged only GABA_A_Rs expressed at the cell surface. We obtained a diffusion coefficient of (10.7 ± 0.9) × 10^−2^ μm^2^/s for α2β2γ1^SEPH^ GABA_A_Rs and (3.2 ± 0.5) × 10^−2^ μm^2^/s for α2β2γ2L^SEPH^ GABA_A_Rs at 32°C, in excellent agreement with the results obtained using mEos2 and with previously published results that quantified the diffusion rate of γ2-containing GABA_A_Rs in hippocampal neurons (Bannai et al., 2009). We therefore conclude that the sptPALM method successfully measured the mobility of GABA_A_Rs inserted in the plasma membrane.

**Figure 6:**
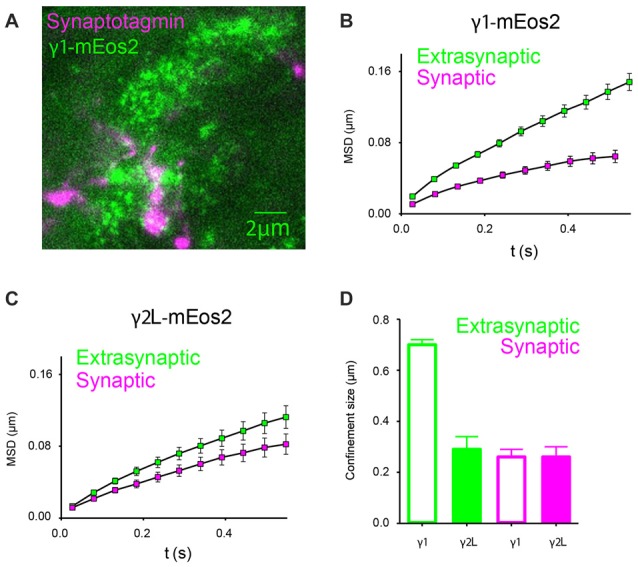
In artificial synapses, α2^SEPH^β2γ2L^mEos2^ GABA_A_Rs diffuse more slowly within synaptic regions. **(A)** Immunolabeling of synaptotagmin-positive presynaptic terminals of cortical neurons (magenta) and a HEK293 cell expressing α2^SEPH^β2γ1^mEos2^ GABA_A_Rs at 32°C (green). Clusters of GABA_A_Rs are visible at the synapse. **(B,C)** Mean MSD vs. time plots for γ1- and γ2-containing GABA_A_Rs obtained from single-channel trajectories within synaptotagmin-labeled regions show stronger confinement and slower diffusion (green squares) compared to those in other parts of the cell membrane where synaptic contacts are not formed (magenta squares). **(D)** Average confinement size in extrasynaptic regions (green) and at synapses (magenta).

In order to examine the mobility of GABA_A_Rs relative to the synapse, we labeled neuronal presynaptic sites with Oyster 650-conjugated synaptotagmin antibodies, and analyzed single-channel trajectories that originated within labeled regions (Figure 6A). At 32°C, α2β2γ1^mEos2^ GABA_A_Rs located at the synapse exhibited an average diffusion coefficient of (2.7 ± 0.5) × 10^−2^ μm^2^/s (*n* = 11), much slower than in extrasynaptic regions (*P* < 0.0001, Student *t*-test). The diffusion rates of synaptic GABA_A_Rs containing γ1 or γ2L subunits were similar (D_α2β2γ2mEos2_ = (2.6 ± 0.9) × 10^−2^ μm^2^/s, *n* = 12). A comparison of MSD curves calculated from synaptic and extrasynaptic regions for γ1- and γ2L-containing receptors is shown in Figures 6B,C. Smaller MSD indicates that the motion of GABA_A_Rs is additionally constrained at the synapse formed between neurons and HEK293 cells. We calculated an average size of the compartments within which GABA_A_R movement was constricted and found that α2β2γ1^mEos2^ receptors diffused within larger domains in extrasynaptic regions of HEK293 cells compared to α2β2γ2L^mEos2^ GABA_A_Rs (0.70 ± 0.02 μm and 0.29 ± 0.05 μm respectively, *P* < 0.0001, Student *t*-test). However, at the synapse both receptor types moved within the domains similar in size; α2β2γ1^mEos2^ and α2β2γ2L^mEos2^ confinement size was 0.27 ± 0.03 μm and 0.26 ± 0.04 μm, respectively (Figure 6D). In addition, γ1- and γ2-containig GABA_A_Rs formed similarly spaced synapses. The mean distance between synaptic clusters was 3.9 ± 0.4 μm and 4.0 ± 0.5 μm for α2β2γ1^mEos2^ and α2β2γ2L^mEos2^ respectively (Supplementary Figure S3A). This result supports the conclusion that differential clustering of receptors cannot explain the differences in IPSC decay rate and provides the first direct evidence for the localization of γ1-containing GABA_A_Rs at synapses.

### Organization of γ1- and γ2-Containing GABA_A_Rs in Neurons

While artificial GABAergic synapses allow us to measure IPSCs produced by GABA_A_R isoforms with defined subunit compositions, they may lack some of the postsynaptic intracellular proteins found in neurons and may therefore not accurately recapitulate the GABA_A_R mobility and clustering properties as observed *in vivo*. To investigate this, we expressed γ1^SEPH^, γ2L^SEpH^ and γ2L^T349L-SEpH^ subunits in cultured cortical neurons and studied their organization and dynamics in synaptic and extrasynaptic regions at 36°C. We used Alexa-647 conjugated anti-GFP antibodies to stochastically label single GABA_A_Rs and Oyster 560-conjugated antibodies to highlight synaptotagmin-rich presynaptic regions. This approach allowed us to create a map of the receptor positions in an uPAINT experiment.

**Figure 7:**
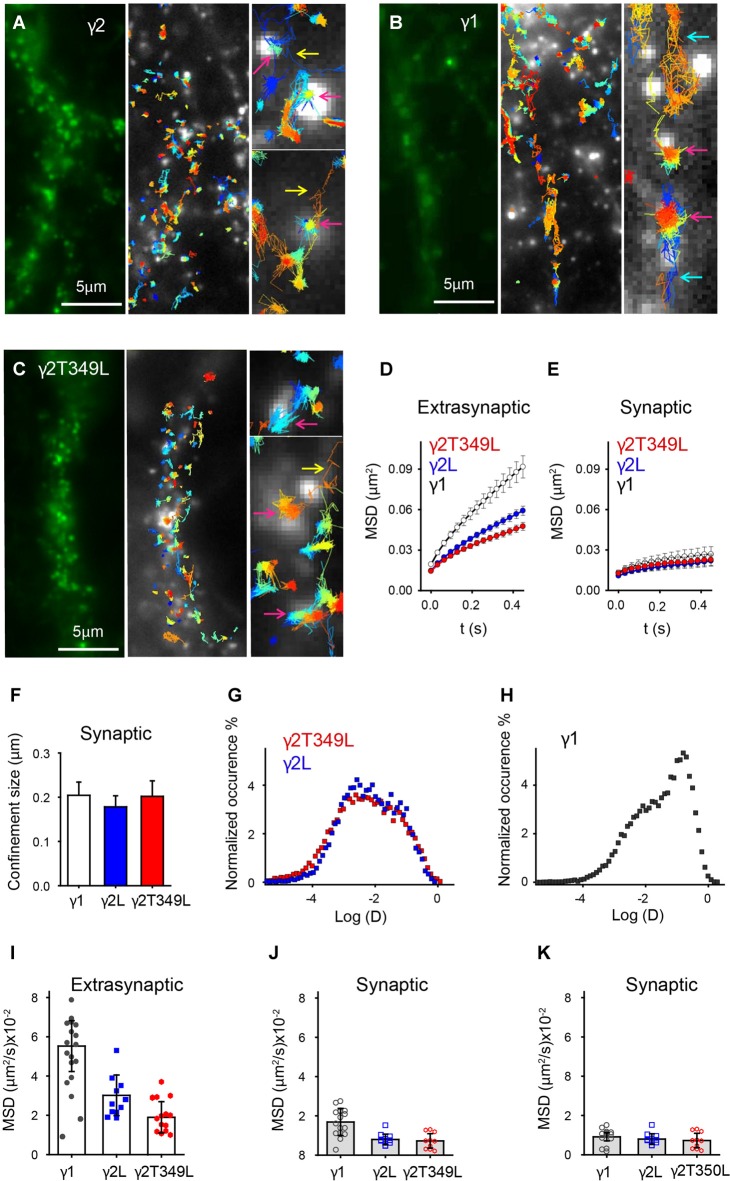
Universal point accumulation for imaging in nanoscale topography (uPaint) imaging in neurons reveals differences in the mobility of GABA_A_Rs with different γ subunits. **(A–C)** Examples of γ2L, γ1 and γ2L^T349L^ subunit organization in cortical neurons. The left panels depict raw images of superecliptic pHluorin (SEpH)-labeled subunits. In uPAINT experiments single GABA_A_Rs are localized in respect to presynaptic regions labeled with synaptotagmin (light puncta in center panels). Zoomed regions in the right panels show examples of the receptors, which are freely diffusing, entering or leaving synaptic regions, or confined at the synapse (turquoise, yellow and magenta arrows, respectively). The width of the zoomed in regions is 1 μm. **(D)** Averaged (±SEM) MSD vs. time plots for extrasynaptic receptors. **(E)** Averaged (±SEM) MSD plot showing that mobility of γ1- and γ2L-containing GABA_A_Rs is similar at synapses. **(F)** Average (±SEM) confinement size of synaptic receptors. **(G,H)** Distribution of instantaneous diffusion coefficients of GABA_A_Rs containing γ2, γ2T349L or γ1 subunits (red, blue and charcoal squares respectively). The distribution is bimodal for all receptors, indicating clustered, less mobile and freely diffusive populations of receptors. For γ1-containing receptors, the peak of the distribution is right shifted, indicating a larger number of freely diffusing receptors. **(I)** A plot showing neuron-to-neuron variability in diffusion coefficients as calculated from the average MSD curve per neuron. **(J)** Same as **(I)** but only synaptic receptors are included. There is no statistical difference between diffusion coefficients. **(K)** Same as **(J)** but the receptors that moved in or out of synaptic regions were excluded from analysis.

GABA_A_Rs containing γ2L subunits formed clusters in cortical neurons (Figure 7A, left panel). As previously described for γ2L-containing GABA_A_Rs (Bannai et al., 2009; Choquet and Triller, 2013), uPAINT revealed a population that diffused freely and a population whose movement was strongly confined within clusters, some of which were in close proximity to synaptotagmin-labeled presynaptic sites (Figure 7A, white puncta, middle panel). Trapping of receptors in synaptic regions and their escape was also evident (Figure 7A, yellow arrows). We classified single-channel trajectories as extrasynaptic or synaptic based on their proximity to presynaptic terminals. GABA_A_Rs were considered to be localized at the synapse if they were found in clusters that overlapped with synaptotagmin labeling (Figure 7A, magenta arrow, right panel) and extrasynaptic otherwise. Similar analysis were performed on γ1 and γ2^T349L^ subunits (Figures 7B,C). As expected, the average MSD of synaptic γ2L-containing GABA_A_Rs was smaller than that of extrasynaptic GABA_A_Rs, consistent with decreased lateral mobility due to the interaction with scaffolding proteins (Figures 7D,E, blue circles). From MSD plots, we calculated that synaptic GABA_A_Rs containing γ2L moved within regions whose size was on average (0.18 ± 0.03) μm (Figure 7F, blue bar).

γ1-containing GABA_A_Rs formed clusters at both synaptic and extrasynaptic sites, but were overall highly mobile, spanning larger distances between clusters than γ2L-containing receptors (Figure 7B). Single-channel trajectories showed that γ1-containing receptors had a higher frequency of exchange between synaptic and perisynaptic populations than γ2L-containing GABA_A_Rs (13.2 ± 1.2%, compared to 7 ± 1% during an average imaging time of 2.5 min). In addition, many γ1-containing receptors visited regions of the neuronal plasma membrane close to synaptotagmin-rich areas but did not dock at the synapse (e.g., Figure 7B, turquoise arrow). The increased mobility of γ1-containing receptors gave rise to the slope of the extrasynaptic MSD curve, which was steeper than that for γ2L-containing GABA_A_Rs (Figure 7D, white circles). However, the γ1-containing receptors localized at the synapse showed similar confined motion to GABA_A_Rs containing γ2L subunits (Figure 7E, Fs, white circles and bar).

The overall shape of the average MSD curve depends on the proportion of clustered receptors with constricted movement, as well as the diffusion coefficients and confinement size. In an attempt to decouple these effects, we plotted a histogram of instantaneous diffusion coefficients obtained from all trajectories for neurons transfected with γ1 or γ2L subunits. Both distributions were bimodal and the range of diffusion coefficients was similar. GABA_A_Rs containing γ2L subunits had the less mobile population dominating the distribution of diffusion coefficients (Figure 7G, blue squares). GABA_A_Rs containing γ1 subunits had a larger fast moving population (Figure 7H, charcoal squares) which increased the MSD of extrasynaptic receptors but did not affect the size of synaptic clusters (Figures 7E,F).

When we expressed γ2L^T349L^, a subunit that switches IPSC kinetics from α2β2γ2L to α2β2γ1, the GABA_A_Rs exhibited motion similar to the γ2L-containing receptors. This is illustrated in Figure 7C, where the correlation between the receptors and synaptotagmin is prominent and the fraction of freely diffusing channels is smaller than that of γ1-containing GABA_A_Rs. The MSD plots, synaptic confinement size and the distribution of instantaneous diffusion coefficients confirmed that the movement of γ2L^T349L^-containing receptors was virtually indistinguishable from that of γ2L-containing GABA_A_Rs (Figures 7E–G, red symbols).

As we did not focus on a specific neuron type, in Figure 7I we compared the cell-to-cell variation in motion of GABA_A_Rs containing different γ subunits by calculating the diffusion coefficients from the averaged MSD curves. As expected, in neurons expressing the γ1 subunit, the measured diffusion coefficients of GABA_A_Rs was significantly higher than that in the receptors expressing γ2L or γ2L^T349L^ subunits (Figure 7I). However, at synaptic sites, the movement of γ1-containing GABA_A_Rs was reduced (Figure 7J). Although not statistically significant, a trend toward higher diffusion coefficients at synaptic sites is a consequence of a higher exchange rate between synaptic and extrasynaptic γ1-containing GABA_A_Rs compared to γ2L- and γ2L^T349L^-containing receptors (Figure 7J, black circles). When we excluded receptors that moved in or out of synaptic regions, the γ1-containing GABA_A_Rs moved as slowly as those containing γ2L subunits (Figure 7K). In addition, we measured absolute distances between synaptic clusters along neurites and found that γ1- and γ2-containing GABA_A_Rs formed synapses at similar distances (3.9 ± 0.3 μm and 4.1 ± 0.5 μm for synaptic clusters containing γ1 or γ2 subunits respectively, Supplementary Figure S3B). Taken together, these results strongly suggest that the difference in IPSC kinetics cannot be explained by changes in flux, residence time or clustering of the receptors at the synapse. Rather, we infer that γ1-containing receptors exhibit slower synaptic current decay due to differential inherent gating dynamics when localized at neuronal synapses.

## Discussion

We sought to understand why, when expressed in artificial synapses, IPSCs mediated by α2β2γ1 GABA_A_Rs exhibit significantly slower decay kinetics than those mediated by α2β2γ2L GABA_A_Rs. Given that the two receptors exhibit similar inherent opening and closing rates when isolated from the synapse (Dixon et al., 2014), we initially hypothesized that the difference in their IPSC decay rates was due to a non-conserved structural element in the large intracellular domain. We identified a single amino acid (Thr349) in the middle of the γ2L subunit cytoplasmic loop that was solely responsible for the differential IPSC decay kinetics. That is, α2β2γ2L^T349L^ receptors exhibited an IPSC decay rate that was α2β2γ1-like, whereas the converse mutation to the γ1 subunit (γ1^L353T^) had the opposite effect. Although Thr349 can be phosphorylated by CaMKII in isolated protein experiments (McDonald and Moss, 1994), replacing Thr349 with phosphomimetic or other residues could not replicate the effect of the T349L mutation. Thus, phosphorylation of Thr349 does not explain the differential kinetics.

We next tested whether the difference in IPSC kinetics was due to an altered propensity of the two GABA_A_R subtypes to cluster at postsynaptic densities. At both glutamatergic and GABAergic synapses delayed neurotransmitter access to extrasynaptic or perisynaptic receptors can lead to synaptic currents with slower rise and decay times (Lozovaya et al., 1999; Oláh et al., 2009; Wu et al., 2012). This mechanism can be revealed by blocking GABA reuptake (Gonzalez-Burgos et al., 2009). However, blocking re-uptake of GABA via GAT-1 inhibition had no effect on the rise or decay times of IPSCs mediated by α2β2γ1 or α2β2γ2L GABA_A_Rs. This could mean that transport is not important at neuron-HEK293 cell synapses, or that IPSC decay at these synapses is determined by the inherent properties of the channels, without opportunities for late-binding channels to slow down the event. Monte Carlo simulations showed that the slow IPSC decay rate at α2β2γ1 synapses cannot be explained by the diffusion of GABA to distant receptors. Spreading receptors further away from the release site did not change IPSC decay kinetics, even when we modeled asynchronous release spread over 5 ms The peak of our IPSCs (both real and simulated) occurred after several milliseconds. In order for delayed binding at distant GABA_A_Rs to contribute to the decay time, new GABA_A_Rs would need to open after the peak, but this is inconsistent with the very short time that it takes for GABA to diffuse out of the cleft (Figure 3A).

In a final attempt to investigate the plausibility of reduced clustering as a mechanism for slow decay, we investigated whether α2β2γ1 and α2β2γ2L GABA_A_Rs exhibited differences in clustering at synapses. In the plasma membrane of HEK293 cells devoid of artificial synaptic contacts, γ1-containing GABA_A_Rs diffused significantly more rapidly than γ2L-containing GABA_A_Rs. In contrast to its effect on IPSC kinetics, the γ2L^T349L^ mutation had no effect on the receptor diffusion rate. However, in the regions where synaptic contacts were formed, γ1-containing GABA_A_Rs were confined to the extent that their mobility was not significantly different to that of γ2L-containing GABA_A_Rs. The diffusion rates for γ2L-containing GABA_A_Rs were similar to those measured previously for α2-containing GABA_A_Rs in neurons (Hausrat et al., 2015). Because the mobility of α2β2γ1 GABA_A_Rs is significantly reduced in postsynaptic regions, we infer that clusters of α2β2γ1 GABA_A_Rs are not displaced from the synapse.

In neurons, γ2L- and γ2L^T349L^-containing GABA_A_Rs both exhibited pronounced clustering, and the global distribution of diffusion coefficients was bimodal. GABA_A_Rs containing the γ1 subunit formed clusters at the synapse and in extrasynaptic regions. In neurons, their diffusion coefficients also displayed a bimodal distribution, however, γ1-containing receptors had larger fast-moving population and higher frequency of escape from clusters compared to γ2L-containing GABA_A_Rs. Considering the tagged γ subunits transfected into neurons are likely to assemble with a range of endogenous α and β subunits, our data from neurons and HEK293 cells correspond remarkably well. Three key features emerge from the pooled results. The first is that γ1-containing GABA_A_Rs have a more pronounced population of freely diffusing receptors in extrasynaptic regions. The second feature is that when γ1-containing GABA_A_Rs are found at synapses they exhibit similar confinement and diffusion rates to γ2L-containing GABA_A_Rs. The third feature is that the γ2L^T349L^ mutation has no effect on receptor mobility, despite its dramatic effect on IPSC kinetics. This last result effectively rules out the possibility that differences in diffusion rates of α2β2γ1 and α2β2γ2L GABA_A_Rs are responsible for the difference in their IPSC decay kinetics.

Neuronal γ2L-containing GABA_A_Rs are found in cholesterol-rich regions and the mobility of both extrasynaptic and synaptic γ2L-containing GABA_A_Rs is accelerated after cholesterol depletion (Renner et al., 2009). There is evidence that cholesterol is enriched in neuronal postsynaptic densities (Renner et al., 2009). Given that γ1-containing receptors have larger flux at the synapse, they might be more likely to reside in low cholesterol regions. This will render γ1-containing GABA_A_Rs more prone to free diffusion, keeping them away from the synapse. In addition to modulating channel mobility, the composition of lipids can impact functional properties of pentameric ligand-gated ion channels through the interaction with the TM4 domain (Barrantes, 2015). However, when we mutated the amino acid residues of γ1 to match those of γ2 in the TM4 domain, the IPSC decay rate was unaffected (Figures 1C,E). Therefore, the change in the IPSC kinetics cannot be explained by the difference in the composition of the lipids surrounding γ1 and γ2 subunit-containing GABA_A_Rs.

In neurons, the mechanisms underlying clustering and mobility of GABA_A_Rs are complex and depend in part on synaptic activity (Bannai et al., 2009). Although GABA_A_Rs may interact with extracellular synaptic proteins (e.g., neurexin Zhang et al., 2010), we restrict our discussion to cytoplasmic proteins, as the mutation that switches IPSC kinetics is located in the large intracellular domain. So far, the only protein known to alter GABA_A_R kinetics is GABARAP (Chen et al., 2000; Luu et al., 2006), which binds to both γ1 and γ2L subunits close to the TM4 domain (Nymann-Andersen et al., 2002), and is thus incompatible with our predicted binding site (Figure 1A). At the synapse, the α2 subunit interacts with the gephyrin-collibystin complex through the large intracellular domain (Saiepour et al., 2010). The same region of γ1 and γ2L subunits is responsible for binding to GABARAP, although this region does not include L353 or T349 residues (Nymann-Andersen et al., 2002). Finally, radixin constrains α5-containing GABA_A_Rs to extrasynaptic regions of the plasma membrane (Hausrat et al., 2015). Out of all these proteins only gephyrin is endogenously expressed in HEK293 cells. However, direct binding of γ1 or γ2L subunits to gephyrin has not been demonstrated. Nevertheless, we cannot reject the possibility that the difference in decay rates is due to a differential interaction between a synapse-specific factor. Indeed recent studies have demonstrated a plethora of proteins at inhibitory synapses, many of which have unknown function (Uezu et al., 2016).

## Conclusion

In conclusion, we have shown that γ1 subunit incorporation in GABA_A_Rs slows the decay rate of IPSCs in artificial synapses and that the Leu353 residue is responsible. Our experiments in both HEK293 cells and neurons identified two novel observations. First, γ1-containing receptors are localized at neuronal synapses, and thus may contribute to fast synaptic inhibition in the brain. At synapses, they exhibit similar confinement and mobilities to γ2L-containing GABA_A_Rs. Second, γ1-containing GABA_A_Rs have larger population of rapidly diffusing extrasynaptic receptors compared to γ2L-containing GABA_A_Rs. High mobility of extrasynaptic γ1-containing GABA_A_Rs facilitates rapid tuning of receptor numbers at the synapse which may be required for homeostatic regulation of network activity in regions of the brain where γ1 subunits are highly expressed (Pirker et al., 2000; Bannai et al., 2009; Esmaeili et al., 2009). As the key residue for altered synaptic kinetics (Leu353) has no effect on synaptic clustering propensities or diffusional properties of receptors, we conclude that this residue is important for synapse-specific alterations in gating properties that differ between γ1- and γ2L-containing GABA_A_Rs.

## Author Contributions

CLD performed the cloning of plasmids, the site-directed mutagenesis and the electrophysiology. AK also performed electrophysiology. ND performed all microscopy measurements. CLD, AK and ND analyzed the data; CLD, ND, JWL and PS conceived the study and all authors contributed to writing the manuscript.

## Conflict of Interest Statement

The authors declare that the research was conducted in the absence of any commercial or financial relationships that could be construed as a potential conflict of interest.
