# Supplementary material for: γ1-Containing GABA-A Receptors Cluster at Synapses Where they Mediate Slower Synaptic Currents than γ2-Containing GABA-A Receptors
Source: Front Mol Neurosci. 2017 Jun 8;10:178. doi: 10.3389/fnmol.2017.00178 (PMC5462899; doi:10.3389/fnmol.2017.00178)
Supplement: Supplementary file 1 [file Image_1.pdf]

Supplementary Information

Supplementary Figure S1

A

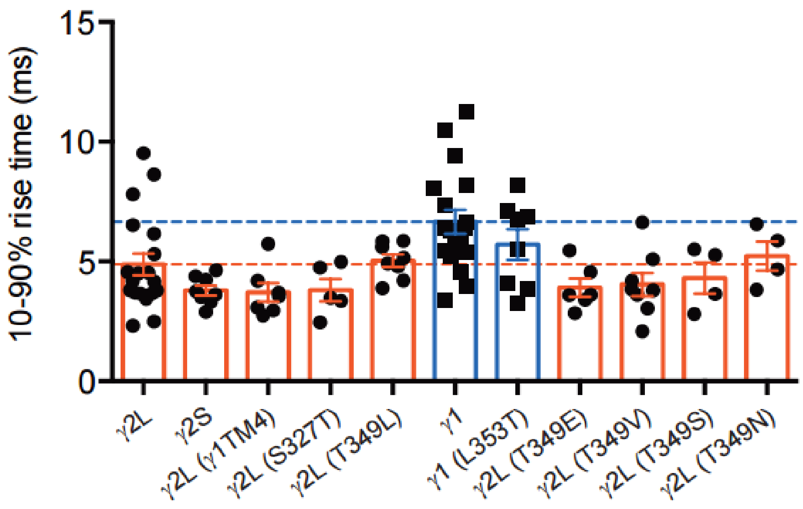

B

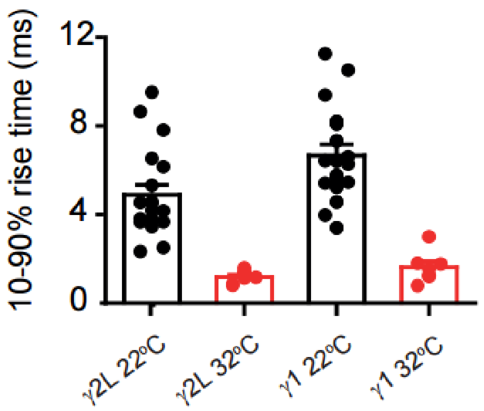

Activation rates of synaptic currents. A. Mean rise time (±SEM) recorded from artificial synapses incorporating the indicated γ subunit variant. Individual data points represent the average of all IPSCs recorded in a single cell. Horizontal lines indicate mean rise time for γ2L (red) and γ1 (blue). IPSCs generated by $GABA_A$Rs containing γ1 subunit have slower rise times than γ2L-containing receptors. **B**. Comparison of IPSC activation rates at 22 °C and 32 °C.

Supplementary Figure S2

A

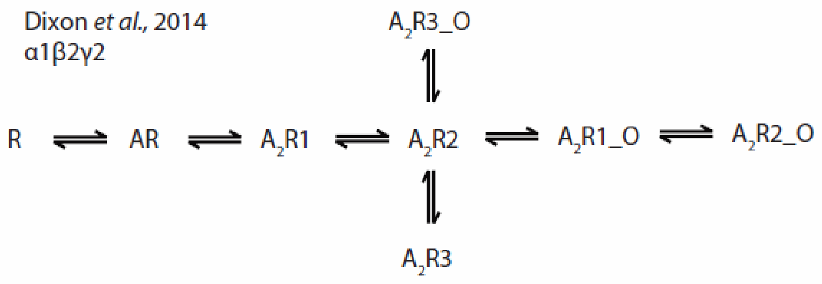

Dixon et al., 2014			Fd	Rv
A' + R'	<->	AR'	[7e+06,	350]
AR' + A'	<->	A2R1'	[3.5e+06,	700]
A2R1'	<->	A2R2'	[1039,	800]
A2R2'	<->	A2R3'	[322.7,	620.8]
A2R2'	<->	A2R1_O'	[1018.3,	1860.2]
A2R1_O'	<->	A2R2_O'	[263.1,	620.1]
A2R2'	<->	A2R3_O'	[960,	881.8]

B

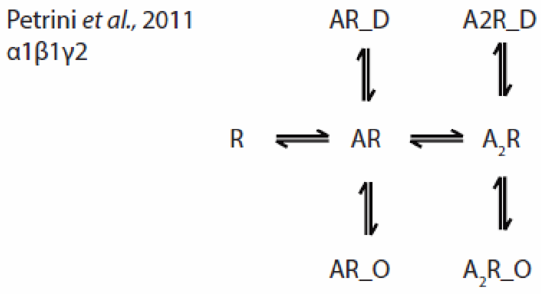

Petrini et al., 2011			Fd	Rv
A' + R'	<->	AR'	[2.87e+06,	200]
A' + AR'	<->	A2R'	[18.27e+06,	400]
AR'	<->	AR_D'	[0.23,	0.35]
AR'	<->	AR_O'	[86,	208]
A2R'	<->	A2R_D'	[1320,	32]
A2R'	<->	A2R_O'	[8850,	420]

Reaction schema highlighting differences between simulation models used in this study. In the model by Dixon et al. (2014), only doubly liganded receptors can transition into open states (**A**), while in the model by Petrini et al. (2011), activation of $GABA_A$Rs by monoliganded states is allowed and binding of subsequent GABA molecule is cooperative (**B**). Units for rate constants are $s^{-1}.M^{-1}$ for binding and s^{-1} for all other transitions.

Supplementary Figure S3

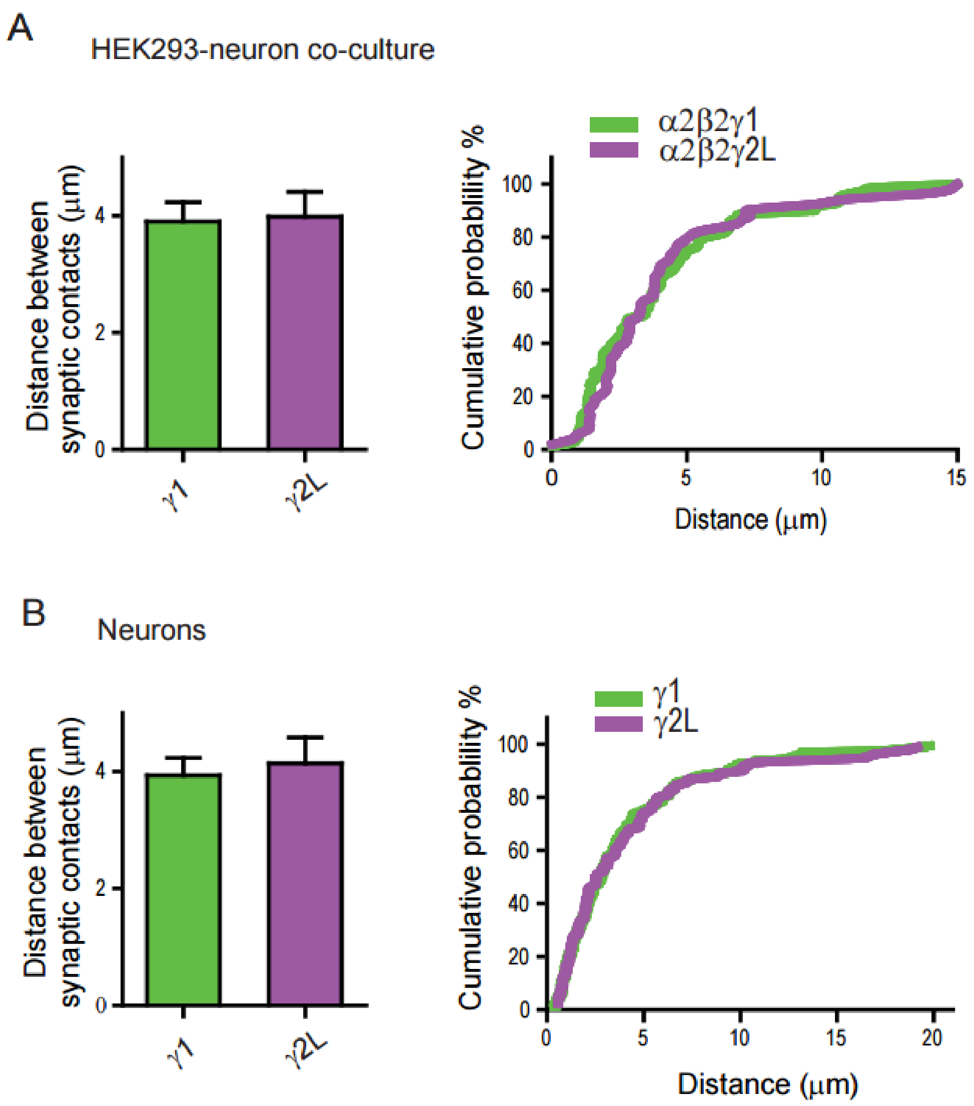

Absolute distances between synapses incorporating γ1**- and** γ2L**-containing receptors are similar.** An average (±SEM) distance among $GABA_AR$ clusters at synaptic contacts formed between cortical neurons and HEK293 cells, and in cortical primary cultures (left panels in **A** and **B** respectively). Cumulative probability confirms that the distribution of distances between the synapses is comparable.
